# Monitoring and management of adverse effects associated with trastuzumab deruxtecan: a UAE-specific consensus

**DOI:** 10.3389/fonc.2024.1443962

**Published:** 2025-01-15

**Authors:** Emad Dawoud, Fathi Azribi, Aref Chehal, Shaheenah Dawood, Syed Hammad Tirmazy, Dina Hamza, Hassan Jaafar, Hussam Marashi

**Affiliations:** ^1^ Department of Oncology, Tawam Hospital, Al Ain, United Arab Emirates; ^2^ Department of Oncology, American Hospital, Dubai, United Arab Emirates; ^3^ Department of Oncology, Sheikh Shakhbout Medical City, Abu Dhabi, United Arab Emirates; ^4^ Department of Oncology, Mediclinic Middle East, Dubai, United Arab Emirates; ^5^ Department of Oncology, Dubai Hospital, Dubai, United Arab Emirates; ^6^ Department of Oncology, Burjeel Hospital, Sharjah, United Arab Emirates

**Keywords:** HER2, metastatic breast cancer, trastuzumab deruxtecan, T-DXd, targeted therapy, antibody-drug conjugates, adverse effects

## Abstract

Breast cancer is the most frequently diagnosed cancer in the UAE and a leading cause of cancer-related mortality. Although early diagnosis contributes to favorable prognoses, novel treatment modalities like antibody-drug conjugates (ADCs) have significantly broadened the therapeutic landscape for patients in metastatic settings. The recognition of “HER2-low” expression as a targetable category has caused a paradigm shift in the management of breast cancer. Although initially developed to target HER2-positive breast cancer, trastuzumab deruxtecan (T-DXd), an ADC, has now also been approved to treat metastatic or unresectable HER2-low breast cancers. Despite the inherent specificity of an ADC, the risk of off-site toxicity exists and is an essential component while assessing the risk-benefit ratio of the treatment. Developing strategies to balance efficacy and safety is crucial, especially for newly approved therapies like T-DXd. Regional perspectives, cultural beliefs, and demographic factors influence treatment decisions and outcomes. The objective of this paper is to establish a UAE-specific consensus among oncologists on practical T-DXd treatment considerations and management of associated side effects. Establishing a consensus on monitoring and managing T-DXd side effects among experts can promote informed decision-making.

## 1 Introduction

Breast cancer is the most common cancer among women globally. According to the World Health Organization (WHO) estimates, in 2022, nearly 2.3 million women were diagnosed with breast cancer, and it was responsible for 670,000 deaths. (1) Breast cancer is also the most frequently diagnosed cancer in the United Arab Emirates (UAE), with a significant majority of cases diagnosed in women less than 50 years old. The median age of diagnosis in the UAE is 48-49 years, and the average age at presentation is a decade earlier than data documented in the West. (2)

In 2017, 834 new cases of breast cancer were diagnosed in the UAE, accounting for 20.23% of all malignant cancer diagnoses. Moreover, it was identified as the leading cause of cancer deaths in women in 2017. (2) The number of newly reported cases in the UAE increased to 1139 in 2021, translating to a crude incidence rate of 40.1 per 100,000 women and an age-standardized incidence rate of about 52 per 100,000. Breast cancer was identified as the most prevalent malignancy among women (36.9% of all female cancer cases) and ranked third in cancer-related mortality (9.64% of annual cancer-related deaths). The age-standardized incidence rates of breast cancer in the UAE have been found to consistently increase from age 25 to about 74, after which they decline drastically. (3)

The prognosis of breast cancer varies considerably depending on the stage at diagnosis, morphological characteristics of the tumor, treatment received, and other specific patient factors. Early diagnosis is crucial for improving outcomes in breast cancer. However, advancements in our understanding of tumor biology and biomarkers, coupled with novel treatment modalities, have significantly expanded the therapeutic options in metastatic settings. The identification of hormone receptors, human epidermal growth factor receptor 2 (HER2), and trophoblast cell-surface antigen 2 (Trop-2) has enabled a personalized selection of treatment for breast cancer. (4–6) Also, the discovery of mutations like BRCA1/2 (breast cancer gene 1 and 2), PIK3CA (phosphatidylinositol-3-kinase [PI3K] catalytic subunit alpha), ESR1 (Estrogen Receptor 1), AKT (protein kinase B), and PTEN (phosphatase and tensin homolog) has further contributed to our ability to tailor treatments and, allowed a more targeted and effective approach to managing breast cancer. (7–11)

‘Targeted therapy’ has reached the next level with the introduction of antibody-drug conjugates (ADCs). Since the approval of the first ADC, gemtuzumab ozogamicin, for the treatment of acute myeloid leukemia in 2000, significant progress in ADC technology over the past two decades has led to FDA approval of 13 ADCs, while over 100 ADCs are currently in various stages of development. (12) Trastuzumab emtansine (T-DM1) that targets the HER2 receptors was the first ADC to be approved by the FDA for the treatment of breast cancer, followed by HER2-targeting and Trop-2-targeting ADCs, trastuzumab deruxtecan (T-DXd) and sacituzumab govitecan (SG), respectively. (13)

ADCs combine specificity and cytotoxicity by linking a cytotoxic drug to a monoclonal antibody (mAb) designed to target specific antigens expressed by cancer cells. This combination allows targeted delivery of the cytotoxic payload to antigen-positive cancer cells, limiting its uptake by normal (antigen-negative) cells. (14)

Despite the inherent specificity of an ADC, the risk of off-site toxicity exists (see **Table 1**). (15) Thus, developing strategies to balance efficacy and safety is crucial, especially for newly approved therapies for which real-world data are still accruing. Comprehensive management of treatment-associated adverse effects is crucial to achieve this balance, minimize treatment interruptions, and optimize patient care.

**Table 1:** Any grade and grade ≥ 3 adverse events associated with T-DXd.

ADC	Target	Adverse events	
		Any Grade	Grade ≥3
Trastuzumab deruxtecan	HER2	Nausea Vomiting Constipation Decreased appetite Diarrhea Constipation Fatigue Alopecia Anemia Neutropenia Leukopenia Thrombocytopenia	Neutropenia Anemia Nausea Leukopenia Lymphopenia Fatigue

T-DXd, Trastuzumab deruxtecan; HER2, Human epidermal growth factor receptor 2.

T-DXd comprises a humanized anti-HER2 mAB (trastuzumab) linked via a tetrapeptide-based cleavable linker to a topoisomerase I inhibitor (deruxtecan). (16) In 2022, the FDA approved it for treating adult patients with unresectable or metastatic HER2-positive breast cancer who had received a prior anti-HER2-based regimen either in the metastatic or in the neoadjuvant or adjuvant setting and whose disease recurred during or within 6 months of completing treatment. (17) This approval was based on the results of DESTINY-Breast03, a multicenter, open-label, randomized phase 3 trial, which showed T-DXd significantly improved overall survival (OS) in this HER2-positive patient cohort compared to T-DM1. (16) However, only around 15–20% of breast cancers express high levels of HER2. (18) And, according to the statistics published by the National Cancer Institute, between 2016 and 2020, HR-positive/HER2-negative was the most prevalent breast cancer subtype diagnosed among US women, followed by HR-negative/HER2-negative, HR-positive/HER2-positive, and HR-negative/HER2-positive subtypes in descending order of prevalence. (19) This rendered nearly 80% of the women, i.e., those with HER2-negative breast cancers, ineligible for treatment with T-DXd.

Interestingly, recent studies have demonstrated that about 60% of metastatic breast cancers categorized as HER2-negative, whether HR-positive or HR-negative, express low levels of HER2 that is — 1) measurable with an immunohistochemistry (IHC) score of 1+ or 2+ and negative *in situ* hybridization (ISH) results and 2) targetable with ADCs like T-DXd. This category is now being defined as “HER2-low” to distinguish it from true HER2-negative (IHC score=0) breast cancers. This distinction has become a crucial aspect in the metastatic breast cancer treatment paradigm after the results of the DESTINY-Breast04 were published. (20) The results showed that compared to the physician’s choice of chemotherapy, T-DXd significantly improved progression-free survival (PFS) (9.9 months vs. 5.1 months, hazard ratio [HR] for disease progression or death=0.50, p<0.001; T-DXd vs. chemotherapy) and OS (23.4 months vs. 16.8 months, HR for death=0.64, p=0.001; T-DXd vs. chemotherapy) in patients with HER2-low breast cancers. This population included both HR-positive and HR-negative breast cancers. In HR-positive, HER2-low patients, constituting nearly 90% of the trial participants, the median PFS with T-DXd and physician’s choice of chemotherapy was 10.1 months and 5.4 months (HR for disease progression or death=0.51, p<0.001), respectively. The OS in the T-DXd and chemotherapy groups was 23.9 months and 17.5 months (HR for death=0.64, p=0.03), respectively. (20)

Thus, the results of this trial and the recognition of “HER2-low” expression as a targetable category have redefined the molecular profiling of breast cancer and caused a paradigm shift in its management.

Although initially developed to target cancers overexpressing HER2, T-DXd is also effective in HER2-low breast cancers, unlike the other HER2-targeting therapy, T-DM1. The efficacy of T-DXd in HER2-low breast cancers has been attributed to the ‘bystander effect’. This mechanism refers to the ability of T-DXd to deliver its cytotoxic payload (deruxtecan) to the HER2-expressing target cells and subsequently to the neighboring cells as well, irrespective of their HER2 expression level.Click or tap here to enter text. (21, 22)

Translating clinical trial data to real-world scenarios involves considering regional perspectives (healthcare infrastructure, regulatory framework, treatment availability, etc.), cultural beliefs (attitudes and perceptions towards treatments), and demographic factors (socioeconomic status, genetic predispositions, etc.), all of which influence treatment decisions and outcomes. As additional clinical trial and real-world data on T-DXd emerge, particularly its role in treating HER2-low breast cancer, establishing a UAE-specific consensus among oncologists on practical treatment considerations and management of associated toxic effects can foster knowledge sharing and informed decision-making. It can help identify the right candidates for T-DXd treatment and facilitate its integration in the HER2-positive and HER2-low metastatic breast cancer treatment algorithm. Most importantly, a regional consensus based on collective expertise can assist oncologists in effectively monitoring, identifying, and managing the adverse effects associated with T-DXd, ensuring that patients most likely to benefit from the therapy are not deprived of it due to unwarranted toxicity concerns.

## 2 Methods

### 2.1 Establishing a consensus on effective monitoring and management of adverse effects associated with T-DXd

Establishing a consensus can ensure that regional perspectives, including considerations related to the healthcare system framework and the unique characteristics of the local patient population, are considered while tailoring treatment and managing adverse effects (AEs) associated with newly approved treatments like T-DXd. With this objective, an expert panel comprising 8 oncologists from the United Arab Emirates (UAE) met on January 29, 2024, in Dubai (UAE) to explore the current practices and share their clinical experiences with T-DXd. Experts with extensive experience in breast cancer management and from diverse healthcare backgrounds (public and private healthcare settings) were selected to participate in this panel. All members of the panel conducted a thorough literature search on the most recent findings related to T-DXd, enabling them to combine the latest evidence with their own clinical experience during the panel discussion. Based on the extensive discussion during this meeting, a survey questionnaire focusing on the place of T-DXd in the treatment algorithm and the management of its adverse effects was designed to establish a consensus among the panelists on their approach in this regard.

### 2.2 Survey design, methodology, and analysis

A modified Delphi survey comprising 24 questions on T-DXd practical treatment considerations and specific adverse effect monitoring and management was emailed to all the panel members. All questions except one were dichotomous, requiring the panelists to answer “yes” or “no.” One question was a ranking question. The questionnaire was designed in a dichotomous (yes/no) format to keep it simple, focused and unambiguous. It was ensured that the questionnaire encompassed aspects in the monitoring and management of T-DXd-related adverse effects that may be overlooked or underemphasized. A stringent, predetermined consensus criterion of >80% agreement was set (consensus was considered achieved if >80% of the panelists voted “yes” on the 23 dichotomous questions). The panelists agreed that disagreements, if any, would be resolved via a second round of discussion and Delphi survey.

## 3 Results

### 3.1 Survey results

Voting on the survey questions was anonymously conducted using Google Forms, allowing the experts to freely express their opinions without feeling compelled to align with the majority. Based on the predefined criteria, a consensus was achieved on all 23 questions (see **Tables 2**–**6**), eliminating the need for a second round of voting. Herein, we elaborate on the rationale for our responses to the survey questions based on published evidence and our clinical expertise.

**Table 2:** Panel consensus on T-DXd treatment approach and its positioning in the HR-positive/HER2-low and HR-negative/HER2-low breast cancer treatment algorithms.

		% of panelists voting “Yes”	Was consensus achieved?
**1**	Do you agree that the response to T-DXd is optimal when used in earlier lines of therapy?	**100**	**Yes**
**2**	Do you agree that in patients with HER2-positive breast cancer, T-DXd should be considered in the second line for eligible patients?	**100**	**Yes**
**3**	Do you agree with the suggested algorithm for HR-positive/HER2-low breast cancer patients shown in **Figure 1A**?	**100**	**Yes**
**4**	Do you agree with the suggested algorithm for HR-negative/HER2-low breast cancer patients shown in **Figure 1B**?	**87.5**	**Yes**
**5**	Do you agree that there is a need for more data on the sequence of ADCs and which other ADCs should be used after treatment with an ADC?	**100**	**Yes**

T-DXd, Trastuzumab deruxtecan; HR, Hormone receptor; HER2, Human epidermal growth factor receptor 2; ADC, Antibody-drug conjugate.

**Table 3:** Panel consensus on the management of nausea, vomiting and diarrhea associated with T-DXd treatment.

		% of panelists voting “Yes”	Was consensus achieved?
Nausea and Vomiting			
**1**	Do you agree that netupitant/palonosetron can be given two hours before T-DXd treatment, followed by a steroid, to prevent the symptoms of nausea and vomiting?	**100**	**Yes**
**2**	Do you agree that olanzapine should be considered if a patient continues to suffer from nausea after taking netupitant/ palonosetron and steroids?	**100**	**Yes**
**3**	Anxiolytic drugs can be given along with treatment with T-DXd if needed. Do you agree?	**100**	**Yes**
**4**	Patients must be informed about the possibility of nausea and vomiting before starting the treatment. This will prevent panic and emergency visits unless there is severe vomiting. Do you agree with this recommendation?	**100**	**Yes**
Diarrhea			
**1**	Do you agree that diarrhea is not much of a concern for patients treated with T-DXd in clinical practice?	**100**	**Yes**

T-DXd, Trastuzumab deruxtecan.

**Table 4:** Panel consensus on the management of alopecia associated with T-DXd treatment.

		% of panelists voting “Yes”	Was consensus achieved?
Alopecia			
**1**	Do you agree that the hair loss caused by T-DXd treatment in HER2-positive and HER2-low patients is temporary and that the hair grows back eventually?	**100**	**Yes**
**2**	Do you agree that alopecia is not a major issue for patients in the metastatic setting, as they are aware of the potential hair loss?	**100**	**Yes**

T-DXd, Trastuzumab deruxtecan; HER2, Human epidermal growth factor receptor 2.

**Table 5:** Panel consensus on the management of interstitial lung disease/pneumonitis associated with T-DXd treatment.

Interstitial Lung Disease (ILD)		% of panelists voting “Yes”	Was consensus achieved?
**1**	Should a baseline CT be a prerequisite for all patients before starting T-DXd treatment to confirm that the patients have no current, suspected, or prior history of ILD/pneumonitis or significant pulmonary disease?	**100**	**Yes**
**2**	Do you agree that asymptomatic grade 1 ILD/pneumonitis is challenging to detect, and physicians should be vigilant about the side effects to prevent a delay in diagnosis?	**100**	**Yes**
**3**	As per your clinical practice, do you agree on thorough history-taking to ascertain whether patients have trouble climbing stairs or are experiencing other respiratory symptoms?	**100**	**Yes**
**4**	Do you agree that patients should be educated about the signs and symptoms of ILD/pneumonitis and T-DXd-related AEs?	**100**	**Yes**
**5**	Do you agree that CT scans are an important radiological modality to assess abnormalities and disease progression and that patients should undergo regular CT scans after starting treatment with T-DXd?	**100**	**Yes**
**6**	Do you agree a multidisciplinary approach should be implemented, with effective collaboration between radiologists, pulmonologists, and the treating physicians?	**100**	**Yes**
**7**	For grade 1 ILD/ pneumonitis, T-DXd should be withheld until the adverse event is fully resolved to grade 0; followed by rechallenge with T-DXd. Do you agree?	**100**	**Yes**

T-DXd, Trastuzumab deruxtecan; CT, Computed tomography; deruxtecan; ILD, Interstitial lung disease; AEs, Adverse effects.

**Table 6:** Panel consensus on the management of fatigue associated with T-DXd treatment.

		% of panelists voting “Yes”	Was consensus achieved?
Fatigue			
**1**	Do you agree that the possible underlying causes of fatigue, such as anemia, psychological conditions, etc., should be assessed?	**100**	**Yes**
**2**	Do you agree that dose reduction helps reduce fatigue and should be considered because treatment is long-term?	**100**	**Yes**
**3**	Do you agree that effective patient counseling is important when starting T-DXd therapy, and that patients should be educated that treatment may cause fatigue in the first cycle and patients may get used to it in the subsequent cycles?	**100**	**Yes**
**4**	Is it important to have a good support network to handle problems such as fatigue, and low-intensity exercises and activities such as yoga and acupuncture can be proposed to help combat fatigue?	**87.5**	**Yes**

T-DXd, Trastuzumab deruxtecan.

### 3.2 General T-DXd treatment considerations

Studies suggest that the clinicopathologic features of breast cancer in Arab patients vary from those seen in the Western patient population and are characterized by early onset of disease, more advanced stage, higher rate of HER2 amplification, and possibly a different somatic mutational profile. (2, 23, 24) Data on the clinicopathologic features specific to the UAE patient population are limited. A study assessing 94 women with breast cancer from the UAE found that 83% of them had invasive ductal carcinoma and were HER2-positive. (2, 25)

Treatment decisions must be guided by international clinical practice guidelines and evidence from clinical trials. In line with the National Comprehensive Cancer Network (NCCN) guidelines for breast cancer treatment, a consensus was achieved by the panelists regarding the overall treatment approach they would use with T-DXd, including its place on the treatment algorithm for HER2-positive, HR-positive/HER2-low, and HR-negative/HER2-low breast cancers (see **Table 2**). (26) However, it is crucial to recognize that these decisions cannot be generalized. Personalized approaches based on prior lines of systemic therapy, including neoadjuvant and adjuvant therapies, would have to be used on a case-by-case basis.

In the HER2-positive patient cohort, the panelists agreed that T-DXd could be considered as a second-line option post-progression on the standard first-line taxane plus trastuzumab/pertuzumab treatment. (26)

**Figure 1:**
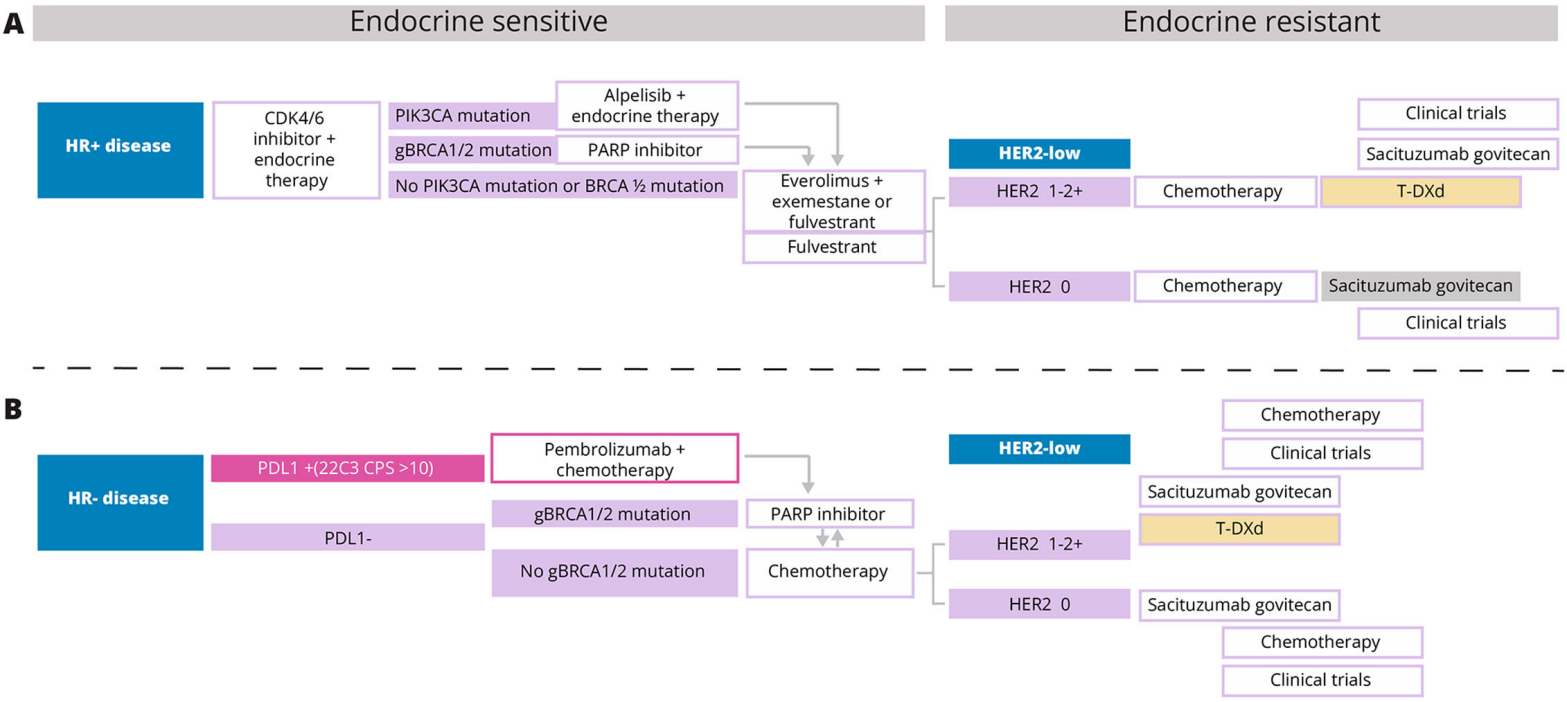
**(A, B)** T-DXd, Trastuzumab deruxtecan; HR, Hormone receptor; HER2, Human epidermal growth factor receptor 2; PDL1, Programmed death ligand 1; PIK3CA, phosphatidylinositol-4,5-bisphosphate 3-kinase catalytic subunit alpha; BRCA1/2, Breast cancer gene 1 and 2.

In the HR-positive/HER2-low cohort, all the panelists agreed (see **Table 2**) with the treatment algorithm (see **Figure 1A**) derived based on the NCCN guidelines and published clinical trial evidence. They agreed that T-DXd could be considered in the second-line setting in endocrine-refractory patients with HR-positive/HER2-low breast cancer who have received at least one prior line of chemotherapy in the metastatic setting or whose disease recurred during or within 6 months of completing adjuvant chemotherapy. Additional lines of chemotherapy or clinical trial enrolment can be considered post-progression on second-line treatment. Although the TROPiCS-02 trial findings have also led to the approval of SG in this setting, the panelists unanimously agreed with preferring T-DXd over SG based on the PFS and OS outcomes of the DESTINY-Breast04 trial. (20, 27, 28) Additionally, the panelists noted that as the patient population enrolled in the DESTINY-Breast04 trial was less pretreated than that of TROPiCS-02, prioritizing T-DXd over SG in this setting is preferable. (20, 27)

The results of the ASCENT trial have paved the way for the approval of SG in the treatment of unresectable locally advanced or metastatic triple-negative breast cancer (TNBC) who have received 2 or more prior systemic therapies, with at least one for metastatic disease. (29, 30) Thus, in patients with HR-negative/HER2-low breast cancers who have progressed after first-line pembrolizumab/chemotherapy, poly ADP-ribose polymerase inhibitors (PARPi) or systemic chemotherapy, both T-DXd and SG may be considered in the second-line setting as detailed within the NCCN guidelines. However, the absence of a head-to-head comparison between these two ADC alternatives makes choosing one over the other challenging and complicates treatment decisions. Based on the published evidence available so far and their expert opinions, seven of eight panelists (87.5%) would consider treatment with T-DXd before SG in HR-negative/HER2-low patients (see **Table 2**), and they agree with the suggested treatment algorithm for HR-negative/HER2-low breast cancers shown in **Figure 1B**. Although based on the predefined criteria consensus was achieved on the proposed treatment algorithm, one panelist did not agree as according to this panelist, the data for the use of SG post-first-line chemotherapy in HR-negative/HER2-low patients are convincing to consider SG before T-DXd in this patient cohort.

### 3.3 T-DXd: specific adverse effect monitoring and management

All the panelists were requested to specify the T-DXd-associated adverse effects they found most challenging to manage to help identify the unique concerns and priorities of oncologists from the UAE. Of the eight panelists, five identified pulmonary toxicity/interstitial lung disease (ILD) as the most challenging adverse effect to manage. Additionally, other adverse effects the panelists usually found challenging to manage included fatigue, nausea, and vomiting. The panelists agreed that hematological toxicities, T-DXd-related neutropenia, and stomatitis can be managed with appropriate supportive therapies.

### 3.4 Gastrointestinal adverse effects

Gastrointestinal adverse effects commonly observed with T-DXd include nausea, vomiting, diarrhea, and constipation. Among these, nausea is the most frequently reported adverse effect, observed in approximately 70% of patients, while the incidence of vomiting is around 40%. (31) Although classified as having moderate emetogenic potential based on the clinical trial data, the NCCN Antiemesis 2024 guidelines have recategorized T-DXd as being highly emetogenic, underscoring the importance of an effective antiemetic prophylactic regimen. (32, 33)

Although nausea/vomiting is reported frequently with T-DXd treatment, in the panelists’ clinical experience, it can be effectively managed with dexamethasone and antiemetics like netupitant, a neurokinin-1 receptor antagonist (NK1 RA), and palonosetron, a serotonin (5-HT3) inhibitor. Based on the 2024 NCCN antiemesis guidelines, the panel agreed that administering netupitant/palonosetron two hours before T-DXd treatment, followed by dexamethasone, can effectively alleviate the symptoms of nausea and vomiting. The panel also recommended including olanzapine in the antiemetic regimen of patients who continue to suffer from nausea/vomiting despite netupitant/palonosetron and dexamethasone treatment. Additionally, an anxiolytic agent can be considered in patients at risk for anticipatory nausea/vomiting. (32) The panel also recommended informing patients about the possibility of nausea/vomiting before initiating treatment as it will help them better manage their expectations and reduce their anxiety and fear (See **Table 3**).

Diarrhea (any grade) was observed in 32% of the patients in the DESTINY-Breast03 trial and 22.4% of the patients in the DESTINY-Breast04 trial. (16, 20) On the other hand, it was one of the most frequent treatment-emergent AEs associated with SG treatment. In the TROPiCS-02 trial, diarrhea (any grade) was observed in 62% of the trial participants treated with SG compared to 23% of those treated with chemotherapy. (27) Unlike with SG, the panel members do not consider diarrhea a significant concern with T-DXd treatment, and it can be managed on a case-by-case basis (See **Table 3**).

### 3.5 Alopecia

Alopecia is a frequent but usually temporary adverse effect associated with T-DXd treatment. (34) In the DESTINY-Breast03 and DESTINY-Breast04 trials, alopecia was observed in around 40% of T-DXd-treated patients. It was the third most frequent treatment-emergent adverse effect in both these trials. (16, 20) While studies have shown scalp hypothermia reduces the risk of alopecia associated with chemotherapy, its efficacy in T-DXd-associated alopecia is not known. (35)

Although alopecia does not affect patients’ physical health, the panel acknowledges that it can adversely impact their psychological and emotional well-being. The panelists recommend counseling patients and providing a supportive environment to address their concerns. They may be offered scalp cooling devices or minoxidil treatment to manage alopecia. However, acknowledging that there is no high-level published literature or surveys on what symptoms matter the most to the patients in the UAE, the panelists do not consider alopecia as a major concern for metastatic breast cancer patients in the country. Moreover, most of them have already experienced alopecia from prior exposure to chemotherapy (see **Table 4**).

### 3.6 Interstitial lung disease/pneumonitis

Drug-induced interstitial lung disease (ILD) or pneumonitis associated with several anticancer therapies is a serious adverse effect that warrants proactive monitoring and prompt treatment to prevent progression to life-threatening complications. ILD has been reported with several classes of anticancer therapies, including chemotherapy, tyrosine kinase inhibitors (TKIs), mammalian target of rapamycin (mTOR) inhibitors, immune checkpoint inhibitors (ICIs), and ADCs, with an incidence rate ranging from 0.4% to 54%. (36) In the DESTINY-Breast03 and DESTINY-Breast04 trials, the incidence of adjudicated drug-induced ILD was 15% and 12.1% of patients treated with T-DXd, respectively, with most cases being classified as either grade 1 or 2. (16, 20)

A pooled analysis of 9 phase I and II T-DXd monotherapy studies that included 1150 heavily pretreated patients with breast, gastric, lung, colorectal, and other cancers revealed an overall incidence of adjudicated ILD/pneumonitis to be 15.4%, with most cases being categorized as low-grade and a median onset time of 5.4 months. This analysis by Powell et al. also showed that patients likely to develop ILD/pneumonitis usually tend to develop it within 12 months from the first T-DXd dose. Although this pooled analysis suggests a favorable benefit-risk ratio, identifying patients at an increased risk of ILD/pneumonitis is essential, and additional studies elucidating the risk factors that may predispose patients to this complication are needed. (37)

All the panelists agreed that baseline computed tomography (CT) imaging is a prerequisite for all patients before initiating T-DXd treatment. CT scans must also be conducted at periodic intervals during treatment with T-DXd to evaluate the development of any abnormalities. CT chest scans must ideally be done at no more than a 12-week interval. Patients should have access to chest imaging without significant delays if they complain of respiratory symptoms. The panelists also recommend a multidisciplinary approach among radiologists, pulmonologists, and oncologists to ensure radiological signs of ILD/pneumonitis are not missed and ILD is treated optimally. This approach is crucial to prevent the progression of grade 1 ILD to a higher grade.

Additionally, detailed history must be collected to assess patients’ respiratory health, and they must be educated to watch for any signs/symptoms of ILD/pneumonitis. The panelists also agreed that grade 1 ILD/pneumonitis is challenging to detect as it is asymptomatic, and oncologists must be vigilant to identify any potential signs to ensure early detection and intervention. T-DXd treatment must be withheld if grade 1 ILD/pneumonitis is diagnosed, and rechallenge may be considered only after its complete resolution (See **Table 5**).

### 3.7 Fatigue

The Common Terminology Criteria for Adverse Events (CTCAE) published by the US Department of Health and Human Services defines fatigue as “A disorder characterized by a state of generalized weakness with a pronounced inability to summon sufficient energy to accomplish daily activities.” According to the CTCAE, grade 1 fatigue is relieved by rest, whereas grade 3 fatigue persists even after rest, causing limitations in self-care activities of daily living like bathing, dressing/undressing, eating, using the toilet, taking medications, etc. (38) In the DESTINY-Breast03 and DESTINY-Breast04 trials, 31% and 47.7% of patients experienced any grade fatigue, and grade ≥ 3 fatigue was reported by 6% and 7.5% of patients in these respective trials. (16, 20)

Fatigue is highly prevalent in the metastatic setting and may be attributed to multiple factors, including direct effects of cancer progression and anticancer treatment side effects. (39) Multiple genetic, psychosocial, and behavioral factors that may increase the risk of fatigue have been identified. They include single-nucleotide polymorphisms (SNPs) in inflammation-related genes, pre-treatment fatigue, depression, sleep disturbances, loneliness, physical inactivity, and elevated body mass index (BMI). (40) Specific personality traits can cause individuals to experience fatigue differently, thus making its assessment highly subjective. (39) Also, it is often underestimated by patients rationalizing it as an inevitable consequence of the cancer or its treatment, and sometimes even by physicians who may focus more on the other acute symptoms and downplay the impact of fatigue. (41)

All the panelists agreed that fatigue significantly impacts cancer patients’ quality of life, and identifying and addressing any underlying potentially treatable causes of fatigue, wherever feasible, is crucial. The panelists also suggested T-DXd dose reduction as an option in patients with severe fatigue, considering that it is a long-term treatment that necessitates balancing efficacy with tolerability. The panelists agreed that counseling patients about all aspects of T-DXd therapy, including potential adverse effects, can help them better manage their expectations and develop coping strategies to manage fatigue. Additionally, providing patients with a robust support network can help alleviate the burden of fatigue. Seven of eight (87.5%) panelists agreed that patients may benefit from incorporating low-intensity exercises like yoga into their daily routines. In some instances, alternative therapies like acupuncture may also be suggested. These non-pharmacological approaches may be utilized to provide additional support to patients in their efforts to combat fatigue (see **Table 6**).

## 4 Discussion

The development of ADCs has opened up several avenues to target cytotoxic payload directly to specific antigen-expressing cancer cells, thus allowing the personalization of treatment approaches based on the molecular profiles of the cancer. ADCs approved so far target antigens overexpressed by cancer cells, such as HER2. However, ongoing research suggests the potential for utilizing antigens expressed by other components of the tumor microenvironment, such as cancer-associated fibroblasts and extracellular matrix, thereby significantly broadening the therapeutic capabilities of ADCs. (14, 42) Exciting possibilities are anticipated in ADC therapy as our understanding of the ADC mechanism of action increases, novel targets are identified, and threshold target levels required for ADC effectiveness are established. As the landscape of ADC therapy evolves, physicians must learn to navigate the different treatment options to select the one that is most likely to be effective while managing the associated toxic effects.

The evolution of T-DXd from a primarily HER2-targeting ADC to one that is also effective against HER2-low cancers represents a significant breakthrough for chemo-refractory HR-negative and HR-positive/HER2-negative breast cancers that fit the HER2-low category and previously had limited therapeutic options. (16, 20, 43) Moreover, an ongoing DESTINY-Breast06 trial is evaluating the efficacy of T-DXd following one or more lines of endocrine therapy in breast cancers with an “ultra-low” level of HER2 expression categorized by IHC > 0 < 1+; IHC 0 no membrane staining. (44–46)

As T-DXd transitions from the highly controlled clinical trial setting to the real world, and its use extends from HER2-positive to HER2-low and may be HER2-ultra low metastatic breast cancer patients (depending on the results of the DESTINY-Breast06 trial), physicians must remain vigilant in monitoring the associated adverse effects and managing them effectively to optimize treatment benefits. (44) The primary goal of conducting this survey was to gather insights from a panel of UAE-based oncologists into the regional perspectives concerning T-DXd treatment and its adverse effects.

The clinical trial evidence supports the use of T-DXd in the second-line setting for metastatic HER2-positive breast cancer patients post-progression on taxane plus trastuzumab/pertuzumab. (16, 17) In the endocrine-refractory HR-positive/HER2-low breast cancer patients, T-DXd can be considered in the second-line setting post-progression on chemotherapy. (20, 47) T-DXd is also approved for the treatment of HR-negative/HER2-low breast cancer post-progression on chemotherapy. (47) However, in this setting, the optimal sequence of the ADCs — T-DXd and SG — remains uncertain in this patient cohort due to the lack of head-to-head comparative studies assessing their safety and efficacy. Additional studies are warranted to address this knowledge gap. In the interim, there was a consensus to prefer T-DXd over SG in the second-line setting in this patient cohort.

Fatigue and ILD/pneumonitis were identified as the most challenging T-DXd-associated adverse effects to manage in the UAE metastatic breast cancer patient population. Diagnosing the precise cause(s) of fatigue is difficult because of its highly subjective nature, multifactorial etiology, and absence of specific diagnostic tests. Nevertheless, due to its adverse impact on patients’ quality of life, it is crucial to inform patients about the possibility of experiencing fatigue, conduct thorough medical evaluations, address the underlying causes, wherever feasible, and suggest non-pharmacological strategies to cope with fatigue.

While ILD/pneumonitis is a serious safety concern associated with T-DXd, proactive monitoring, diagnosis, and treatment with the support of a multidisciplinary team comprising oncologists, pulmonologists, and radiologists can mitigate this risk. Educating patients to recognize and promptly report symptoms of ILD/pneumonitis can facilitate diagnosis. (48, 49) The identification of predictive biomarkers is urgently needed to assess ILD risk, enabling stratification of patients based on their risk level and customization of monitoring and management strategies accordingly. (48) Current evidence suggests a rechallenge with T-DXd should be considered only after the complete resolution of grade 1 ILD. (45, 48, 49) According to the prescribing instructions, permanent discontinuation of T-DXd is recommended in all patients diagnosed with grade 2 or higher ILD. (50) Studies evaluating the risks and benefits of rechallenging in this patient population and identifying potential predictors for ILD recurrence can assist in informed clinical decision-making.

## 5 Conclusions

The findings of this study indicate that the T-DXd treatment approach used by oncologists in the UAE is consistent with the international guideline recommendations. The survey conducted in this study did not identify any novel safety concerns associated with T-DXd in the UAE patient population. Fatigue was recognized as the most bothersome symptom and one that is challenging to manage. ILD/pneumonitis is a serious adverse effect that warrants proactive and multidisciplinary management strategies. Other T-DXd-associated adverse effects like nausea, vomiting, hematological toxicities, neutropenia, and stomatitis can be managed with appropriate therapies.

The results of this survey have laid the foundation for developing more comprehensive, UAE-specific protocols that multidisciplinary teams can use to monitor and manage T-DXd-associated adverse effects. Implementing these protocols can help prevent unnecessary treatment discontinuation and ensure patients receive the full benefit of T-DXd treatment while minimizing the risk of adverse effects.

## Data Availability

The raw data supporting the conclusions of this article will be made available by the authors, without undue reservation.
